# Association between oxidative balance score and new-onset hypertension in adults: A community-based prospective cohort study

**DOI:** 10.3389/fnut.2022.1066159

**Published:** 2022-12-15

**Authors:** Jun-Hyuk Lee, Da-Hye Son, Yu-Jin Kwon

**Affiliations:** ^1^Department of Family Medicine, Nowon Eulji Medical Center, Eulji University School of Medicine, Seoul, South Korea; ^2^Department of Medicine, Graduate School of Hanyang University, Seoul, South Korea; ^3^Department of Family Medicine, Gangnam Severance Hospital, Yonsei University College of Medicine, Seoul, South Korea; ^4^Department of Family Medicine, Yongin Severance Hospital, Yonsei University College of Medicine, Yongin-si, South Korea

**Keywords:** oxidative balance score, oxidative stress, hypertension, cohort study, Korean

## Abstract

**Introduction:**

Oxidative stress plays a key role in the pathophysiology of hypertension development. The oxidative balance score (OBS) comprises dietary and lifestyle pro- and anti-oxidant components and reflects the overall oxidative stress burden. We aimed to evaluate the association between the OBS and new-onset hypertension (HTN) using large, community-based, prospective Korean cohort data.

**Methods:**

Among 10,030 participants aged 40–69 years included in the Korean Genome and Epidemiology Study, the data of 5,181 participants were analyzed. The hazard ratio (HR) and 95% confidence interval (CI) for new-onset HTN according to sex-specific OBS quartile groups were calculated using univariable and multivariable Cox proportional hazard regression analyses.

**Results:**

During the mean 13.6-year follow-up period, 1,157 men and 1,196 women developed new-onset HTN. Compared to the Q1 group, the adjusted HRs (95%CI) for new-onset HTN in the Q2, Q3, and Q4 groups were 0.96 (0.82–1.16), 0.85 (0.72–0.99), and 0.71 (0.59–0.86) in men and 0.81 (0.69–0.95), 0.81(0.68–0.95), and 0.70 (0.57–0.84) in women, respectively.

**Discussion:**

Individuals with high OBS are at lower risk of developing HTN. This study suggests that a healthy lifestyle and antioxidant rich diet could be a preventive strategy for HTN.

## Introduction

Hypertension (HTN) is the most important modifiable cardiovascular risk factor. It is associated with significant morbidity and mortality, and has become an international health concern (1, 2). Globally, the number of individuals with HTN nearly doubled, from 648 million in 1990 to 1,278 million in 2019 and in the United States, more than 670,000 deaths had hypertension as a primary or contributing cause in 2020 (3, 4). It is well known that continuously elevated blood pressure leads to serious complications, including myocardial infarction, stroke, and death (5, 6). Therefore, early detection and prevention of HTN is important to reduce the burden of major cardiovascular diseases (CVDs).

Although the pathogenesis of HTN is complex and multifactorial, persuasive evidence has shown that oxidative stress—defined as an imbalance between pro- and anti-oxidant components—is one of the fundamental mechanisms responsible for the development of HTN (7, 8). Oxidative stress not only damages macromolecules and deoxyribonucleic acid, but also disrupts redox signaling, leading to abnormal cell signaling (9, 10). Under physiological conditions, several modulators interact to maintain normal blood pressure, including the renin-angiotensin system (RAS), sympathetic nervous system, natriuretic peptide, sodium excretion, and endothelial (11). Oxidative stress causes dysfunction and damage to these regulators and organs that regulate blood pressure, leading to high blood pressure (12, 13). For example, oxidative stress such as oxygen free radical O_2_- and superoxide anion rapidly degrade vasodilating factor NO, causing vasoconstriction (14). Also, the prevalence of hypertension is significantly higher in men than in premenopausal women, and it is known that the relatively higher oxidative stress burden in men may cause sexual dimorphism in hypertension (15). In several animal studies, antioxidants such as apocynin and tempol decreases blood pressure in male, whereas female animal are non-responsive (16, 17). Moreover, the oxidative stress mechanism is associated with the development of various diseases, such as CVD, diabetes, cancer, inflammatory disorders, and Alzheimer's disease (18). However, unlike animal, human studies have yielded inconsistent results regarding the impact of oxidative stress on health (19, 20). This discrepancy among studies may be attributed to the extremely complex interactions of multiple pro- and anti-oxidant factors with endogenous enzymatic mechanisms and exogenous factors. Various exogenous factors, including diet, smoking, exercise, and medicines, affect oxidative stress; therefore, the oxidative balance score (OBS) was developed to reflect the overall oxidative stress burden using dietary and lifestyle pro- and anti-oxidant components (21). Antioxidant components contribute positively, whereas pro-oxidant components contribute negatively; therefore, lower OBS values reflect higher pro-oxidant exposures. The OBS has been validated in several studies involving cancer, chronic kidney disease, and CVDs (22–25). Although there is evidence regarding an inverse association of OBS with HTN prevalence (26), there is lack of evidence regarding the association of OBS with new-onset HTN. If a significant relationship between OBS and incident HTN is proven, then managing OBS could be a cost-effective strategy to prevent the development of HTN. Therefore, we aimed to evaluate the association between OBS and the risk of new-onset HTN in Korean middle-aged and older adults using community-based, large-scale Korean cohort data.

## Methods

### Participants

**Figure 1:**
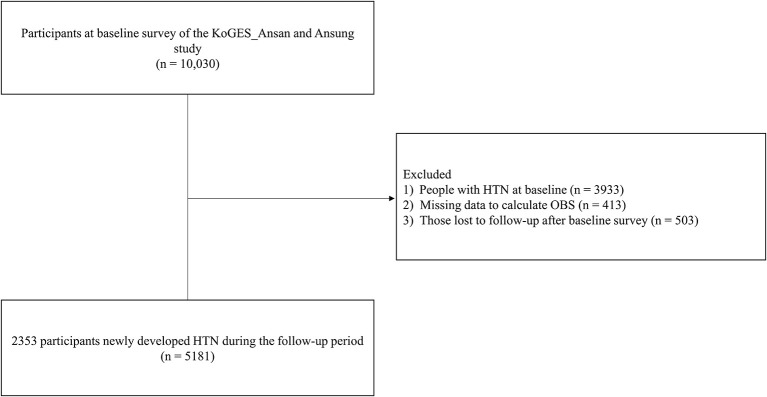
Flowchart of the study population. KoGES, korean genome and epidemiology study; HTN, hypertension; OBS, oxidative balance score.

We used data from the Korean Genome and Epidemiology Study (KoGES)_Ansan and Ansung study. The current cohort study was conducted within the KoGES, the detailed design of which was previously described (27). During the KoGES_Ansan and Ansung study, a total of 10,030 adults aged 40–69 years were included in the baseline survey (2001–2002) and followed up every 2 years up to 2017–2018. Participants were excluded if they had HTN at baseline (*n* = 3,933), missing OBS data (*n* = 413), or were lost to follow-up after the baseline survey (*n* = 503). A total of 5,181 participatns were included in the final analysis (men: 2,386; women: 2,795). The study flow chart is shown in Figure 1.

The KoGES_Ansan and Ansung cohort protocol was reviewed and approved by the institutional review board (IRB) of the Korea Centers for Disease Control and Prevention. Informed consent was obtained from all participants. The study protocol conformed to the guidelines of the 1964 Declaration of Helsinki and its later amendments. This study was approved by the institutional review board of Nowon Eulji Medical Center (IRB Number: 2021-09-025).

### Assessment of oxidative balance score

The OBS comprised 15 components including seven pro-oxidant and six anti-oxidant factors based on previous studies (23, 25, 28, 29). The OBS assignment scheme was described in Table 1. There were eight pro-oxidant factors as follows: saturated fatty acid (SFA), omega-6 poly-unsaturated fatty acid (PUFA), total iron intake, total copper intake, and statuses pertaining to smoking, drinking, obesity, and abdominal obesity. The seven anti-oxidant factors were as follows: omega-3 PUFA, vitamin C, vitamin E, selenium, beta-carotene, zinc, and physical activity. Each question was scored 0, 1, or 2 except for abdominal obesity status, which was scored as 0 or 1. The scores for SFA, omega-6 PUFA, total iron intake, and total copper intake were assigned as low (2 points), intermediate (1 point), and high (0 points) according to the sex-specific tertile values of each variable. For smoking status, the scores for never-, former-, and current smoker were assigned 2, 1, and 0 points, respectively. For drinking status, non-, mild to moderate- and heavy drinkers were assigned 2, 1, and 0 points, respectively. For obesity status, obesity, overweight, and normal were assigned 0, 1, and 2 points, respectively. Abdominal obesity and normal were assigned 0 and 1 point, respectively. The scores for omega-3 PUFA, vitamin C, vitamin E, selenium, beta-carotene, and zinc intake were assigned as low (0 points), intermediate (1 point), and high (2 points), according to the sex-specific tertile values of each variable. For physical activity, low physical activity, moderate physical activity and high physical activity were assigned to 0, 1, and 2 points, respectively. The OBS ranged from 0 to 29 points. We divided the participants into sex-specific quartiles according to the OBS (Q1, lowest quartile; Q2, second quartile; Q3, third quartile; and Q4, highest quartile groups).

**Table 1:** Oxidative balance score assignment scheme.

**OBS components**	Assignment scheme^*^
1. Saturated fatty acid (P)	0 = high (3rd tertile), 1 = intermediate (2nd tertile), 2 = low (1st tertile)
2. omega-6 PUFA intake (P)	0 = high (3rd tertile), 1 = intermediate (2nd tertile), 2 = low (1st tertile)
3. Total iron intake (P)	0 = high (3rd tertile), 1 = intermediate (2nd tertile), 2 = low (1st tertile)
4. Total copper intake (P)	0 = high (3rd tertile), 1 = intermediate (2nd tertile), 2 = low (1st tertile)
5. Smoking status (P)	0 = current smoker, 1 = former smoker, 2 = never smoker
6. Drinking status (P)	0 = heavy drinker (≥30 g/day in men, ≥20 g/day in women), 1 = mild-to-moderate drinker (<30 g/day in men, <20 g/day in women), 2 = non-drinker
7. Overweight/obese (P)	0 = obese, 1 = overweight, 2 = normal weight
8. Abdominal obesity (P)	0 = abdominal obesity, 1 = normal
9. omega-3 PUFA intake (A)	0 = low (1st tertile), 1 = intermediate (2nd tertile), 2 = high (3rd tertile)
10. Vitamin C intake (A)	0 = low (1st tertile), 1 = intermediate (2nd tertile), 2 = high (3rd tertile)
11. Vitamin E intake (A)	0 = low (1st tertile), 1 = intermediate (2nd tertile), 2 = high (3rd tertile)
12. Selenium intake (A)	0 = low (1st tertile), 1 = intermediate (2nd tertile), 2 = high (3rd tertile)
13. Total beta-carotene intake (A)	0 = low (1st tertile), 1 = intermediate (2nd tertile), 2 = high (3rd tertile)
14. Zinc intake (A)	0 = low (1st tertile), 1 = intermediate (2nd tertile), 2 = high (3rd tertile)
15. Physical activity (A)	0 = low (<7.5 METs-hr/wk), 1 = moderate (7.5–30 METs-hr/wk), 2 = high (>30 METs-hr/wk)

* Low, intermediate, and high categories correspond to sex-specific tertile values among participants in the KoGES at the baseline survey.

P, pro-oxidant; A, anti-oxidant; PUFA, poly-unsaturated fatty acid; MET, metabolic equivalent of task; KoGES, korean genome and epidemiology study.

### Definition of hypertension

HTN was defined as follows: systolic blood pressure (SBP) ≥140 mmHg, or diastolic blood pressure (DBP) ≥90 mmHg, or treatment with anti-hypertensive drugs.

### Covariates

Participants underwent health examinations and interviews by well-trained medical staff. Body mass index (BMI) was defined as the body mass divided by the height squared (kg/m^2^). BMI ≥ 25 kg/m^2^ was defined as obese, and BMI ≥ 23 kg/m^2^ as overweight, according to the 2018 Korean Society for the Study of Obesity (KSSO) guideline (30). Waist circumference (WC, cm) was measured at the midpoint between the iliac crest and the lowest rib. Abdominal obesity was defined when the waist circumference was ≥90 cm in men and ≥85 cm in women, according to the 2018 KSSO guideline (30). Smoking status was classified as follows; never-, former-, and current smoker. The alcohol consumption status was classified as: non-drinker, mild to moderate drinker (individual drinking <30 g/day alcohol in men and < 20 g/day in women), heavy drinker (individual drinking ≥30 g/day alcohol in men and ≥20 g/day in women). Physical activity was measured as metabolic equivalent of task (MET)-hours per week (MET-h/week) using the International Physical Activity Questionnaire (31). Physical activity was classified as low (<7.5 MET-h/week), moderate (7.5–30 METs-h/week), and high physical activity (>30 MET-h/week). Menopausal status was defined as having no periods for at least 12 months. Nutritional status was assessed using the food frequency questionnaire (FFQ) *via* a face-to-face interview conducted by a well-trained dietitian at each participant's home (32). Daily total energy intake (kcal/day), SFA (g/day), omega-6 PUFA, total iron (mg/day), omega-3 PUFA (g/day), vitamin C (mg/day), vitamin E (mg/day), selenium (μg/day), copper(μg/day), zinc (mg/day), and beta-carotene (μg/day) were assessed in this study. Each dietary intake was calculated by computer aided nutritional analysis program (CAN-pro) 5.0 software (Seoul, Korea), which is the most widely used program for nutrition counseling in Korea (33). Education level was classified as below elementary school, middle school, high school, and college or above. Monthly household income was categorized into three groups: <100 million, 100–200 million, and >200 million Korean Won. The blood sample of each participant was obtained after at least 8 h fasting. The blood glucose, serum insulin, total cholesterol, triglyceride, high density lipoprotein (HDL) cholesterol, and C-reactive protein (CRP) levels were measured using a Hitachi 700-110 Chemistry Analyzer (Hitachi, Ltd., Tokyo, Japan).

### Statistical analysis

All statistical analyses were performed after testing for normality. Data are presented as means ± standard deviations or medians (25th, 75th interquartile ranges) for continuous variables or as a number (percentages, %) for categorical variables. Differences among quartile groups were compared using analysis of variance or the Kruskal–Wallis test for continuous variables, and the chi-square test for categorical variables. Inverse Kaplan–Meier curves with the log-rank test were used to determine the cumulative rates of incident HTN according to sex-specific OBS quartile groups.

We performed univariable and multivariable Cox proportional hazard regression analyses to calculate the hazard ratio (HR) and 95% confidence interval (CI) for incident HTN according to OBS quartiles. In the adjusted model, we adjusted for age, education level, monthly household income, total energy intake, fasting plasma glucose (FPG), serum total cholesterol, and serum CRP levels. All statistical analyses were conducted using SAS statistical software (version 9.4; SAS Institute Inc., Cary, NC, USA) and R (version 4.0.3; R Foundation for Statistical Computing, Vienna, Austria). The significance level was set at *p* < 0.05.

## Results

The baseline characteristics of the study population across sex-specific OBS quartile groups are summarized in Table 2. In men, the Q1 group exhibited the highest mean values for SBP, DBP, serum glucose, total cholesterol, triglyceride, and CRP levels, whereas the Q4 group possessed the highest mean value for serum HDL cholesterol and total energy intake. In women, the Q1 group displayed the highest mean values for age, SBP, DBP, FPG, total cholesterol, triglyceride, and CRP, whereas the Q4 group had the highest mean values for serum HDL cholesterol and total energy intake. The proportions of participants with the highest education level and those with the highest household incomes were significantly higher in the Q4 group.

**Table 2:** Baseline characteristics of the study population.

	**Oxidative balance scores**									
	**Men**					**Women**				
**Variables**	**Q1 (*n* = 717)**	**Q2 (*n* = 655)**	**Q3 (*n* = 607)**	**Q4 (*n* = 407)**	p^*^	**Q1 (*n* = 638)**	**Q2 (*n* = 742)**	**Q3 (*n* = 783)**	**Q4 (*n* = 632)**	p^*^
Age, years	49.7 ± 8.1	50.9 ± 8.6	50.6 ± 8.6	50.1 ± 8.7	0.327	51.8 ± 8.7	50.3 ± 8.6	50.3 ± 8.3	48.3 ± 7.7	<0.001
SBP, mmHg	115.6 ± 10.2	115.2 ± 10.0	114.6 ± 10.5	114.5 ± 10.3	0.032	114.1 ± 11.2	113.2 ± 11.4	113.1 ± 11.5	111.6 ± 11.5	<0.001
DBP, mmHg	78.1 ± 6.8	77.7 ± 6.3	76.9 ± 6.8	76.6 ± 7.5	<0.001	75.8 ± 7.4	74.7 ± 7.4	74.8 ± 7.5	73.7 ± 7.8	<0.001
Glucose, mg/dL	89.9 ± 22.7	88.6 ± 21.3	89.1 ± 23.1	85.3 ± 15.4	0.003	83.7 ± 15.5	83.5 ± 19.3	82.5 ± 14.2	82.0 ± 16.5	0.030
Total cholesterol, mg/dL	193.3 ± 34.4	191.5 ± 34.9	189.0 ± 34.7	185.2 ± 33.0	<0.001	193.1 ± 36.1	188.5 ± 34.2	185.6 ± 32.6	181.4 ± 30.3	<0.001
Triglyceride, mg/dL	163.0 (117.0;232.0)	136.0 (103.5;187.5)	129.0 (95.0;180.5)	117.0 (92.0;164.5)	<0.001	129.0 (98.0;177.0)	119.0 (88.0;161.0)	113.0 (88.0;156.0)	105.0 (82.0;141.5)	<0.001
HDL cholesterol, mg/dL	41.9 ± 9.1	43.7 ± 9.9	43.9 ± 10.0	44.6 ± 10.7	<0.001	44.7 ± 9.4	45.9 ± 10.0	46.4 ± 9.6	47.5 ± 10.0	<0.001
CRP, mg/dL	0.2 ± 0.4	0.3 ± 0.8	0.2 ± 0.4	0.2 ± 0.3	0.033	0.2 ± 0.3	0.2 ± 0.4	0.2 ± 0.3	0.2 ± 0.5	0.107
Menopausal status						318 (49.8%)	313 (42.2%)	352 (45.0%)	209 (33.1%)	<0.001
Education level, *n* (%)					0.524					<0.001
Elementary/middle school	265 (37.0%)	267 (40.9%)	216 (35.8%)	155 (38.3%)		429 (67.8%)	462 (62.8%)	453 (58.1%)	334 (53.0%)	
High school	274 (38.2%)	238 (36.4%)	249 (41.2%)	154 (38.0%)		167 (26.4%)	225 (30.6%)	263 (33.7%)	221 (35.1%)	
College/university	178 (24.8%)	148 (22.7%)	139 (23.0%)	96 (23.7%)		37 (5.8%)	49 (6.7%)	64 (8.2%)	75 (11.9%)	
Household income, *n* (%)					0.050					<0.001
<100 million Korean Won	152 (21.3%)	170 (26.2%)	136 (22.6%)	111 (27.3%)		249 (40.1%)	244 (33.1%)	239 (31.2%)	186 (29.9%)	
100–200 million Korean Won	231 (32.4%)	181 (27.8%)	196 (32.5%)	136 (33.4%)		184 (29.6%)	226 (30.6%)	222 (28.9%)	177 (28.4%)	
>200 million Korean Won	329 (46.2%)	299 (46.0%)	271 (44.9%)	160 (39.3%)		188 (30.3%)	268 (36.3%)	306 (39.9%)	260 (41.7%)	
Energy intake, kcal/day	27.4 ± 28.9	25.1 ± 31.0	22.2 ± 29.5	19.6 ± 29.6	<0.001	5.5 ± 10.0	4.8 ± 9.2	4.6 ± 7.7	3.2 ± 3.7	0.021

* p-value for the comparison of the baseline characteristics among sex-specific quartile groups of oxidative balance score at the baseline survey.

Significance was set at p < 0.05.

SBP, systolic blood pressure; DBP, diastolic blood pressure; HDL, high-density lipoprotein; CRP, C-reactive protein.

Clinical characteristics regarding individual OBS components are shown in Table 3. In both men and women, mean values of SFA, omega-6 PUFA, total iron, omega-3 PUFA, vitamin C, vitamin E, selenium, beta-carotene, and zinc intake increased according to the increasing sex-specific OBS quartile. The proportions of current smokers, heavy drinkers, obese participants, participants with abdominal obesity and participants with low physical activity were significantly higher in the sex-specific Q1 group. Also, proportion of obese participants (63.8%) and participants with abdominal obesity (48.6%) were higher in women compared with men (51.3% for obese and 26.4% for abdominal obesity).

**Table 3:** Individual components of the score by oxidative balance scores quartile.

	**Oxidative balance scores**									
	**Men**					**Women**				
**Variables**	**Q1 (*n* = 717)**	**Q2 (*n* = 655)**	**Q3 (*n* = 607)**	**Q4 (*n* = 407)**	p^*^	**Q1 (*n* = 638)**	**Q2 (*n* = 742)**	**Q3 (*n* = 783)**	**Q4 (*n* = 632)**	p^*^
Saturated fatty acid, g/day	8.7 ± 3.9	10.5 ± 6.2	12.4 ± 6.8	13.8 ± 6.6	< 0.001	8.0 ± 3.8	9.4 ± 5.7	11.7 ± 6.7	15.1 ± 10.1	<0.001
omega-6 PUFA intake, g/day	7.6 ± 4.3	8.3 ± 4.9	9.0 ± 5.6	9.3 ± 4.7	<0.001	7.4 ± 3.9	7.9 ± 5.5	9.1 ± 5.5	9.8 ± 6.1	<0.001
Total iron intake, mg/day	16.0 ± 6.0	18.7 ± 8.5	22.8 ± 11.2	24.8 ± 9.5	<0.001	14.6 ± 5.3	16.5 ± 7.0	21.2 ± 10.0	26.0 ± 13.5	<0.001
Copper intake, μg/day	1,154.5 ± 858.4	1,215.1 ± 891.8	1,438.9 ± 956.4	1,382.4 ± 740.2	<0.001	1,219.2 ± 961.6	1,158.0 ± 848.3	1,448.4 ± 951.3	1,550.1 ± 932.0	<0.001
Smoking status, *n* (%)					<0.001					<0.001
Current smoker	480 (66.9%)	348 (53.1%)	272 (44.8%)	126 (31.0%)		45 (7.1%)	28 (3.8%)	12 (1.5%)	4 (0.6%)	
Former smoker	165 (23.0%)	198 (30.2%)	193 (31.8%)	115 (28.3%)		15 (2.4%)	8 (1.1%)	6 (0.8%)	2 (0.3%)	
Never smoker	72 (10.0%)	109 (16.6%)	142 (23.4%)	166 (40.8%)		578 (90.6%)	706 (95.1%)	765 (97.7%)	626 (99.1%)	
Drinking status, *n* (%)					<0.001					<0.001
Heavy drinker	197 (27.5%)	124 (18.9%)	90 (14.8%)	33 (8.1%)		18 (2.8%)	14 (1.9%)	6 (0.8%)	0 (0.0%)	
Mild to moderate drinker	400 (55.8%)	341 (52.1%)	318 (52.4%)	159 (39.1%)		218 (34.2%)	229 (30.9%)	194 (24.8%)	120 (19.0%)	
Non-drinker	120 (16.7%)	190 (29.0%)	199 (32.8%)	215 (52.8%)		402 (63.0%)	499 (67.3%)	583 (74.5%)	512 (81.0%)	
Obesity status, *n* (%)					<0.001					<0.001
Obese	368 (51.3%)	235 (35.9%)	152 (25.0%)	54 (13.3%)		407 (63.8%)	304 (41.0%)	268 (34.2%)	85 (13.4%)	
Overweight	198 (27.6%)	186 (28.4%)	187 (30.8%)	100 (24.6%)		162 (25.4%)	210 (28.3%)	212 (27.1%)	193 (30.5%)	
Normal weight	151 (21.1%)	234 (35.7%)	268 (44.2%)	253 (62.2%)		69 (10.8%)	228 (30.7%)	303 (38.7%)	354 (56.0%)	
Abdominal obesity, *n* (%)	189 (26.4%)	115 (17.6%)	54 (8.9%)	17 (4.2%)	<0.001	310 (48.6%)	213 (28.7%)	189 (24.1%)	71 (11.2%)	<0.001
omega-3 PUFA intake, g/day	1.1 ± 0.7	1.3 ± 0.8	1.5 ± 0.9	1.6 ± 0.8	<0.001	1.0 ± 0.6	1.2 ± 0.9	1.4 ± 0.9	1.7 ± 1.1	<0.001
Vitamin C intake, mg/day	73.3 ± 38.5	103.6 ± 74.9	142.9 ± 103.6	171.1 ± 108.2	<0.001	75.0 ± 50.5	108.6 ± 85.6	159.0 ± 137.7	221.1 ± 156.3	<0.001
Vitamin E intake, mg/day	10.5 ± 3.8	13.5 ± 6.6	17.0 ± 9.3	19.2 ± 7.2	<0.001	9.1 ± 3.3	11.6 ± 6.1	16.0 ± 7.9	20.8 ± 11.1	<0.001
Selenium intake, μg/day	37.5 ± 18.3	49.6 ± 31.5	62.5 ± 36.6	72.1 ± 31.4	<0.001	30.2 ± 17.1	39.5 ± 23.0	54.6 ± 32.2	73.9 ± 53.1	<0.001
Beta-carotene intake, μg/day	2,228.9 ± 1,729.1	3,301.6 ± 2,880.8	4,646.9 ± 4,304.3	5,338.5 ± 3,714.7	<0.001	1,916.9 ± 1,269.8	2,629.6 ± 1,893.9	4,250.4 ± 3,515.0	5,793.2 ± 4,690.9	<0.001
Zinc intake, mg/day	10.7 ± 2.9	12.2 ± 4.6	14.2 ± 5.2	15.5 ± 4.4	<0.001	10.0 ± 3.0	11.0 ± 3.9	13.5 ± 5.2	16.0 ± 6.4	<0.001
Physical activity, *n* (%)					<0.001					<0.001
Low (<7.5 METs-hr/wk)	73 (10.2%)	36 (5.5%)	28 (4.6%)	6 (1.5%)		98 (15.4%)	67 (9.0%)	53 (6.8%)	37 (5.9%)	
Moderate (7.5–30 METs-hr/wk)	507 (70.7%)	399 (60.9%)	375 (61.8%)	204 (50.1%)		421 (66.0%)	516 (69.5%)	522 (66.7%)	370 (58.5%)	
High (>30 METs-hr/wk)	137 (19.1%)	220 (33.6%)	204 (33.6%)	197 (48.4%)		119 (18.7%)	159 (21.4%)	208 (26.6%)	225 (35.6%)	

* p value for the comparison of the baseline characteristics among sex-specific quartile groups of oxidative balance score at the baseline survey.

Significance was set at p < 0.05.

PUFA, poly-unsaturated fatty acid; MET, metabolic equivalent of task.

**Figure 2:**
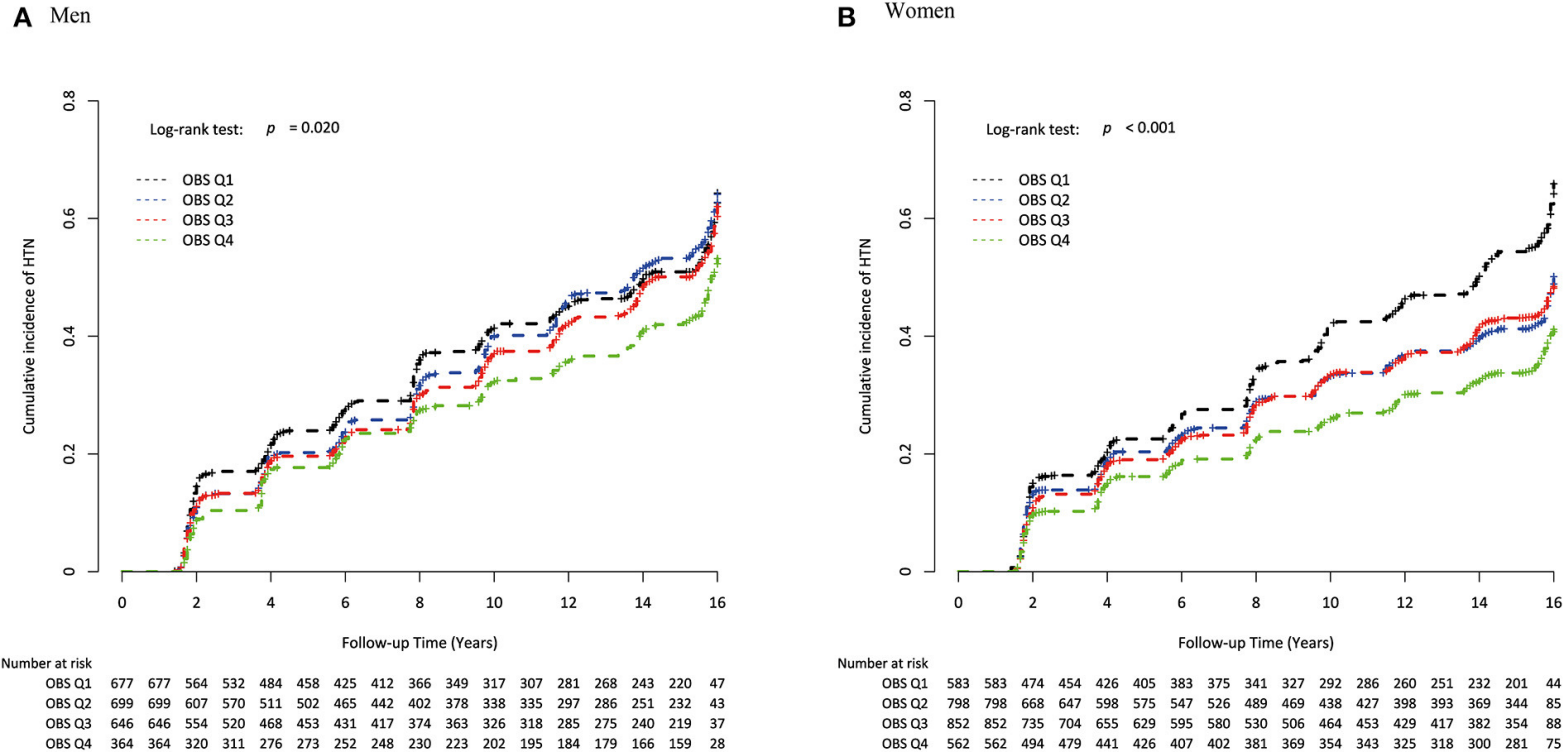
Inverse Kaplan-Meier plot of incident HTN by sex-specific OBS quartile in **(A)** men and **(B)** women. HTN, hypertension; OBS, oxidative balance score.

**Figure 3:**
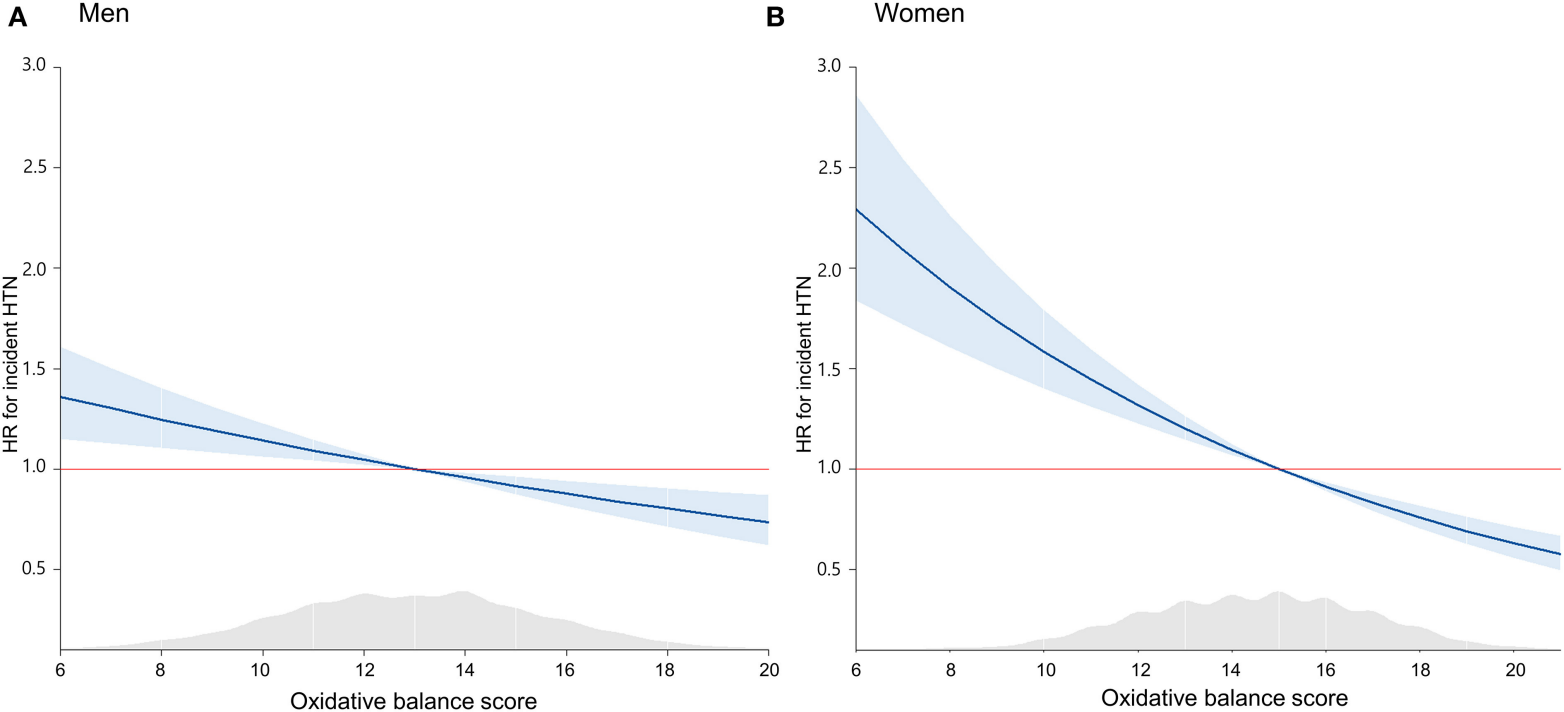
Spline curve for the cox proportional hazard model of incident HTN by sex-specific OBS in **(A)** men and **(B)** women. HTN, hypertension; OBS, oxidative balance score.

During the mean follow-up period of 13.6 years, a total of 1,157 men and 1,196 women developed new onset HTN. The incidences of new-onset HTN during follow-up are presented in Supplementary Table 1. Inverse Kaplan–Meier curves revealed the highest risk of cumulative HTN incidence in the Q1 group, followed by Q2, Q3, and Q4 groups in both men and women (Figure 2), with significant differences (both log-rank test *p* < 0.001). Table 4 shows the independent relationship between OBS and incident HTN, using the HR and 95% CI. Compared to the Q1 group, the HRs (95% CI) for incident HTN in Q2, Q3, and Q4 groups were 1.04 (0.89–1.20), 0.94 (0.81–1.10) and 0.82 (0.68–0.98) in men, and 0.76 (0.65–0.89), 0.78 (0.67–0.91), and 0.62 (0.53–0.74) in women, respectively. In the adjusted model, the adjusted HRs (95%CI) for new-onset HTN in Q2, Q3, and Q4 vs. Q1 were 0.96 (0.82–1.16), 0.85 (0.72–0.99), and 0.71 (0.59–0.86) in men and 0.81 (0.69–0.95), 0.81 (0.68–0.95), and 0.70 (0.57–0.84) in women, respectively. The adjusted HRs and 95% CI for new-onset HTN for each increment of 1 in the OBS were 0.94 (0.92–0.97) in both men and women. Figure 3 showed that incident HTN was negatively associated with OBS in dose-dependent manner.

**Table 4:** Cox proportional hazard regression analysis showing the relationship of oxidative balance scores with incident non-alcoholic fatty liver disease.

**Oxidative balance score quartiles**	**Numbers, n**	**New-onset cases, *n***	**Follow-up period, person-year**	**Incidence rate per 1,000 person-years**	**Unadjusted**	**Adjusted**
					**HR (95% CI)**	**HR (95% CI)**
**Men**						
Continuous (per 1 increment)					0.96 (0.94–0.98)	0.94 (0.92–0.97)
Q1	717	357	6,848.8	52.13	1 (reference)	1 (reference)
Q2	655	333	6,226.3	53.48	1.04 (0.89–1.20)	0.96 (0.82–1.16)
Q3	607	286	5,897.9	48.49	0.94 (0.81–1.10)	0.85 (0.72–0.99)
Q4	407	181	4,243.9	42.65	0.82 (0.68–0.98)	0.71 (0.59–0.86)
*p* for trend					0.023	<0.001
**Women**						
Continuous (per 1 increment)					0.91 (0.89–0.94)	0.94 (0.92–0.97)
Q1	638	330	6,307.1	52.32	1 (reference)	1 (reference)
Q2	742	309	7,702.3	40.12	0.76 (0.65–0.89)	0.81 (0.69–0.95)
Q3	783	327	8,038.9	40.68	0.78 (0.67–0.91)	0.81 (0.68–0.95)
Q4	632	230	6,976.3	32.97	0.62 (0.53–0.74)	0.70 (0.57–0.84)
*p* for trend					<0.001	<0.001

Adjusted for age, education level, monthly household income, total energy intake, fasting plasma glucose, serum total cholesterol, and serum C-reactive protein levels.

HR, hazard ratio; CI, confidence interval.

## Discussion

In this prospective cohort study, we found that lower OBS at baseline was associated with an increased risk of incident HTN in both men and women. Our result agrees with previous findings of human and animal experiments. Reduced endogenous anti-oxidant activity has been widely reported in hypertensive animal models and humans, suggesting that impairment of antioxidant defenses is associated with HTN (34–36). Also, several studies demonstrated that various exogenous anti-oxidants are inversely associated with blood pressure (37, 38). Anti-oxidant treatment to decrease oxidative stress burden prevented the development of HTN in a rat model (37). A large-scale cohort study conducted in France revealed that a high anti-oxidant diet was inversely associated with the risk of incident HTN in women (38).

The most well-known dietary interventions for the prevention of HTN are the Dietary Approaches to Stop Hypertension (DASH) diet and the Mediterranean diet which are characterized by high consumption of vegetable, fruits, and fish or sea foods (39).

Meanwhile, calorie restriction and intermittent fasting that do not take into account the diet quality are known to reduce oxidative stress (40). Also, the effect of antioxidant supplements on blood pressure has not been convincing (41–44). An intervention trial conducted in China revealed that the antioxidant supplementation group exhibited a lower risk of HTN among men after 6 years of follow-up, but not among women (45). In addition, a randomized primary prevention study did not identify an association between new-onset HTN and anti-oxidant supplementation (46). Although the exact reasons for discrepancy between these results and our findings are unclear, we considered only antioxidants (omega-3 PUFA, vitamin C, vitamin E, selenium, beta-carotene, zinc) from foods on the OBS. The inconsistent effects of antioxidant supplements may be due to the lack of consideration of pharmacokinetic properties and the complex mechanism between anti- and pro-oxidant factors.

In this regard, the use of a combination of multiple oxidative stress-related factors to assess the overall oxidative stress burden appears to be more strongly associated with health outcomes than individual factors. To date, there is one cross-sectional study that used OBS to examine the relationship with HTN in 317 participants. In this study, OBS was inversely associated with hypertension, however none of four oxidative stress biomarkers was significantly associated with hypertension after adjustment. Compared with the low OBS group, the adjusted odds ratio (95% CI) of the high OBS group was 0.17 (0.03–0.95) for HTN (26). Although this study is significant in that it is the first study to reveal the relationship between OBS and HTN, there is a limitation that a causal interpretation is not warranted due to the cross-sectional design. To our knowledge, our study is the first to demonstrate an inverse association between OBS and the risk of incident HTN using cohort data.

The type and number of components comprising the OBS varies across studies. According to a review published in 2019, the number of OBS components varied from 3 to 20 (28). Among various anti-oxidant components, almost all OBSs included vitamin C, vitamin E, and beta-carotene and a considerable proportion of OBSs included selenium, lycopene, and lutein. Pro-oxidant components included alcohol and iron intake most often, followed by PUFAs and SFAs (28). Although other environmental factors, such as radiation, xenobiotics, air and water pollutants are also important pro-oxidant components, there were no study included these factors in OBS due to data limitation.

In this study, we used OBS comprising eight pro-oxidant components (SFA, omega-6 PUFA, total iron intake, total copper intake, smoking status, drinking status, obesity, and abdominal obesity) and seven anti-oxidant components (omega-3 PUFA, vitamin C, vitamin E, selenium, beta-carotene, zinc, and physical activity). However, our findings for several pro-oxidant components were inconsistent with previous studies. With increasing sex-specific OBS quartiles, the mean values for SFA, omega-6 PUFA, and total iron intake increased while those of other pro-oxidant components decreased. This inconsistency may be due to the effect of total energy intake that also tended to increase according to increasing OBS quartiles. Also, since the OBS is defined as the total oxidative stress burden, individual components may show independent trends.

The results of this study were significant for both men and women. The adjusted HRs of Q4 compared to Q1 was 0.71 (0.59–0.86) for men and 0.70 (0.57–0.84) for women, but adjusted HRs of Q2 compared to Q1 was 0.96 (0.82–1.16) and 0.81(0.69–0.95), showing lower HRs in women. This result suggests that sex differences exist in the physiological mechanism of oxidative stress. A previous study suggested that females possess a greater antioxidant potential compared to males (17), possibly due to sex differences in the expression and activity of antioxidant enzymes and sex hormones (47). Especially, estradiol decreases expression and activity of NADPH oxidase, which generates reactive oxygen species, whereas it increases antioxidant enzymes such as superoxide dismutase and glutathione peroxidase (48). In addition, phenolic hydroxyl group of estrogen contribute to its antioxidant role by scavenging free radicals (49). After menopause, increased oxidative stress burden medicated by reduced estradiol production increases the incidence of CVDs in postmenopausal women (50). In this regard, this study suggests that more anti-oxidant lifestyles and diets are needed to lower the risk of HTN in men without the antioxidant potential of estradiol than in women.

The current study has several limitations. First, recall and selection bias may exist in the FFQ. Second, all the components of OBS were equally weighted. Since every component has different pro- or anti-oxidant potency, equal-weights for all OBS components may not appropriately reflect the actual biological contributions. Nevertheless, previous studies revealed no significant difference between weighted and unweighted OBS for association with risk of prostate cancer and other endpoints (23, 25, 51). Third, the OBS does not include endogenous factors (gut microbiomes and genes related to antioxidant enzymes) and environmental factors (air and water pollutants, Ultraviolet-B radiation, pathogen infection, and extreme temperature) that modify oxidative stress. Moreover, the OBS was determined under the assumption that the properties of all components exerted a linear effect on oxidative stress, but this did not take into account the threshold effects of anti-oxidants that could exhibit toxic pro-oxidant activity at higher doses or under particular conditions (52). Finally, since we only have baseline FFQ data, we could not analyze changes of OBS over time. Despite these limitations, we believe that our finding of an inverse association between OBS and incident HTN in a large population-based prospective cohort study is informative. Other large prospective studies are required to confirm our results.

## Conclusion

Individuals with high OBS are at lower risk for the development of HTN among community-dwelling middle-aged and older Korean adults. This study suggests that a healthy lifestyle and diet rich in antioxidants to increase the OBS may help prevent HTN in both men and women.

## Data availability statement

Data analyzed in this study were obtained from the Korean Genome and Epidemiology Study (4851–302) and are available at the following website: https://nih.go.kr/eng/main/main.do.

## Ethics statement

The studies involving human participants were reviewed and approved by Institutional Review Board of the Korea Centers for Disease Control and Prevention. The patients/participants provided their written informed consent to participate in this study.

## Author contributions

D-HS, Y-JK, and J-HL: study concept and design, acquisition, analysis, and interpretation of data, drafting the manuscript, and approval of the final manuscript. Y-JK and J-HL: study concept and design, interpretation of data, supervision, and revising the manuscript.
