# Supplementary material for: Association between oxidative balance score and new-onset hypertension in adults: A community-based prospective cohort study
Source: Front Nutr. 2022 Dec 15;9:1066159. doi: 10.3389/fnut.2022.1066159 (PMC9798298; doi:10.3389/fnut.2022.1066159)
Supplement: Supplementary file 1 [file Table_1.DOC]

**Table S1. Incidence of hypertension during the follow-up study**

| Year range | Follow-up | Total (n) | Incidence cases (n) | Incidence rate per 2 years |
| --- | --- | --- | --- | --- |
| 2001–2002 | Baseline | 5181 |  |  |
| 2003–2004 | 2 years | 4828 | 694 | 14.37 |
| 2005–2006 | 4 years | 4268 | 309 | 7.24 |
| 2007–2008 | 6 years | 3818 | 210 | 5.50 |
| 2009–2010 | 8 years | 3863 | 300 | 7.77 |
| 2011–2012 | 10 years | 3628 | 212 | 5.84 |
| 2013–2014 | 12 years | 3487 | 187 | 5.36 |
| 2015–2016 | 14 years | 3713 | 210 | 5.66 |
| 2017–2018 | 16 years | 3628 | 231 | 6.37 |
